# The Number Needed to Treat: 25 Years of Trials and Tribulations in Clinical Research

**DOI:** 10.5041/RMMJ.10218

**Published:** 2015-07-30

**Authors:** Samy Suissa

**Affiliations:** Centre for Clinical Epidemiology, Lady Davis Institute—Jewish General Hospital, Montreal, Quebec, Canada; and Department of Epidemiology and Biostatistics, McGill University, Montreal, Quebec, Canada

**Keywords:** Biostatistics, effect measures, epidemiologic methods, observational studies, randomized controlled trials, treatment impact

## Abstract

The number needed to treat (NNT) is a simple measure of a treatment’s impact, increasingly reported in randomized trials and observational studies, but often incorrectly calculated in studies involving varying follow-up times. We discuss the NNT in these contexts and illustrate the concept using several published studies. While the computation of the NNT is founded on the cumulative incidence of the outcome, several published studies use simple proportions that do not account for varying follow-up times, or use incidence rates per person-time. We show how these approaches can lead to erroneous values of the NNT and misleading interpretations. For example, a trial of 3,845 very elderly hypertensives randomized to a diuretic or placebo reported a NNT of 94 treated for 2 years to prevent one stroke, though the correct approach results in a NNT of 63. Also, meta-analyses involving trials of differing lengths often report a single NNT, such as the meta-analysis of 22 trials of the anticholinergic tiotropium in chronic obstructive pulmonary disease that reported a NNT of 16 patients “over one year,” even if the trials varied in duration from 3 to 48 months, with the actual NNTs varying widely from 15 to 250. Finally, we describe the value of the NNT in assessing benefit–risk, such as low-dose aspirin use in secondary prevention of mortality assessed against the risk of gastrointestinal bleeding. As the “number needed to treat” becomes increasingly used in the comparative effectiveness and safety of therapies, its accurate estimation and interpretation become crucial to avoid distorting clinical, economic, and public health decisions.

## INTRODUCTION

The number needed to treat (NNT) is a simple and user-friendly measure of the impact of a treatment on a given disease outcome, introduced over 25 years ago and now used extensively in randomized trials and observational studies.^1^,^2^ It quantifies the number of patients that need to be treated with the drug or intervention under study in order to prevent disease outcome in one patient. A published example, using pooled data from a meta-analysis of six randomized controlled trials of secondary prevention of cerebrovascular and cardiovascular thrombotic events, reported that the number needed to treat with low-dose aspirin to prevent one death was 67.^3^ This implies that for every 67 patients with stroke, TIA, myocardial infarction, or a history of angina, treatment with low-dose aspirin will prevent one death compared to no treatment for the same 67 patients.

The simplicity of this measure, both in terms of calculation and interpretation, has led to its increasing use in clinical research and prominent presence in publications. However, its incorrect computation, despite or perhaps because of its simplicity, has led to inappropriate and misleading conclusions.^2^,^4^ For example, the above-reported number needed to treat of 67 patients with low-dose aspirin does not specify how long the treatment should last to prevent one death: 1 month, 1 year, 20 years?

In this paper, we review the “number needed to treat” measure and present some examples of its misuse, generally related to the inappropriate consideration of time. We also describe the proper calculation techniques and its accurate interpretation.

## BASICS OF THE NNT MEASURE

The NNT is based on the frequency of disease outcome measured as a cumulative incidence of the outcome per number of patients followed over a given time period. This will result in a proportion of patients with the outcome, which we mechanically tend to write as a percentage, such as 0.5/100, 2/100, or 5/100, to make frequencies easily comparable. Instead of using percentages to facilitate comparisons, we could alternatively express the proportions by fixing the numerator at 1. In our example these become 1/200, 1/50 and 1/20, which when inverted to 200:1, 50:1, and 20:1 represent the number of patients that need to be observed to find one patient with the outcome. This reversed representation of the proportion forms the basis for the NNT.

The NNT relates to the effectiveness of a treatment, usually based on a randomized or observational trial comparing two treatment alternatives, measured by the treatment difference in the proportion of patients with the adverse disease outcome over a fixed follow-up time-period. This difference will represent the proportion of patients for whom the adverse outcome was prevented due to treatment. Inverting this difference will produce the number of patients that need to be treated to prevent one patient with the outcome.^1^

For example, consider a trial of the effectiveness of adding a new inhaler to the usual maintenance treatment of chronic obstructive pulmonary disease (COPD) compared with placebo, where 15% of patients on placebo had a fatal exacerbation compared with 10% with the new inhaler during the 1-year follow-up. Thus 15%–10%=5% represents a prevented mortality of 5/100, that is five patients are prevented from a fatal exacerbation for every 100 patients who added the new inhaler over 1 year instead of adding placebo. The NNT is then simply 1/(15%–10%)=100/5=20. This inversion suggests that rather than using the 5/100 measure of prevented mortality, the NNT value of 20 will provide the more vivid interpretation of one patient avoiding the fatal outcome for every 20 patients treated for 1 year.

A reality of most randomized trials or observational studies is that the follow-up is not equal for all patients but varies, so that the calculation of the proportion with the outcome by the end of the follow-up will require consideration of this variation in follow-up times. In this case, the Kaplan–Meier approach must be used to estimate the correct proportions with the outcome over time; it accounts for varying follow-up times and provides a curve for the cumulative incidence over time.^5^ The NNT is then computed by inverting the difference in the cumulative incidence of the outcome between the two groups, at the desired time of follow-up.^6^ This NNT will represent the number of patients that need to be treated to prevent one patient with the outcome over the given desired time period. This approach has been properly used in several recent trials.^7^–^12^

## EXAMPLES OF PROBLEMATIC NNTS

The long-acting anticholinergic tiotropium and the fluticasone-salmeterol combination are two popular treatments in COPD. A meta-analysis of 22 tiotropium randomized trials involving over 23,000 COPD patients reported, in comparison with placebo, a NNT of “16 patients over one year with tiotropium to prevent one exacerbation.”^13^ On the other hand, the TORCH randomized trial of 6,000 COPD patients reported a NNT of “4 patients over one year with the fluticasone-salmeterol combination in comparison with placebo to prevent one exacerbation.”^14^ This enormous difference in the NNT is puzzling in view of the practically comparable effectiveness of these two treatments in preventing exacerbations.^15^

A randomized trial of the effect of adding zoledronic acid to endocrine therapy in premenopausal women with endocrine-responsive early breast cancer involved 1,803 patients who were followed for up to 84 months.^16^ The follow-up varied, with a median of 48 months. The authors reported that “the number needed to treat with zoledronic acid to prevent disease progression in 1 patient was 31 at a median follow-up of 47.8 months.” The NNT was based on the simple proportion of patients whose disease progressed despite the fact that follow-up varied extensively between patients.

A randomized trial of treatment for herpes simplex virus HSV type 2 (HSV-2) was conducted where 1,484 subjects were randomly assigned to the nucleoside analogue valacyclovir (*n*=743) or placebo (*n*=741) for 240 days.^17^ The authors stated that “one would expect to treat 38 persons with recurrent genital herpes for a year to prevent one case of HSV-2 infection.” Here again, the NNT was based on simple proportions despite the fact that follow-up varied between patients. Moreover, the trial followed patients up for a maximum of 240 days. How valid is it then to extrapolate the NNT from the 240-day study period to 1 year? Can we safely assume that the drug effect extends equally beyond 240 days?

A trial of 3,845 very elderly hypertensives who were randomized to a diuretic or placebo was conducted, with mean follow-up of 2.1 years varying from 0 to 6.5. The outcome was stroke, and the NNT was computed as “1 stroke being prevented because 94 patients were treated for 2 years.”^18^ Can the NNT be applied to the mean follow-up time when this time varies so widely from 0 to 6.5? What about treatment for different periods of time such as 1, 5, or 6 years?

## ILLUSTRATION: HYPOTHETICAL RANDOMIZED TRIAL DATA

To illustrate the analytic issues related to the NNT, data from a hypothetical trial were generated, involving patients with a (imaginary) serious form of iron overload syndrome (IOS), which results in liver failure in almost 50% of patients within a year of diagnosis. A total of 3,000 patients were studied in this hypothetical three-arm trial, with 1,000 randomized to a treatment called Fedom, 1,000 randomized to another treatment called Feclad, both compared with 1,000 patients given placebo, and all patients followed for 1 year or until liver failure.

**Figure 1:**
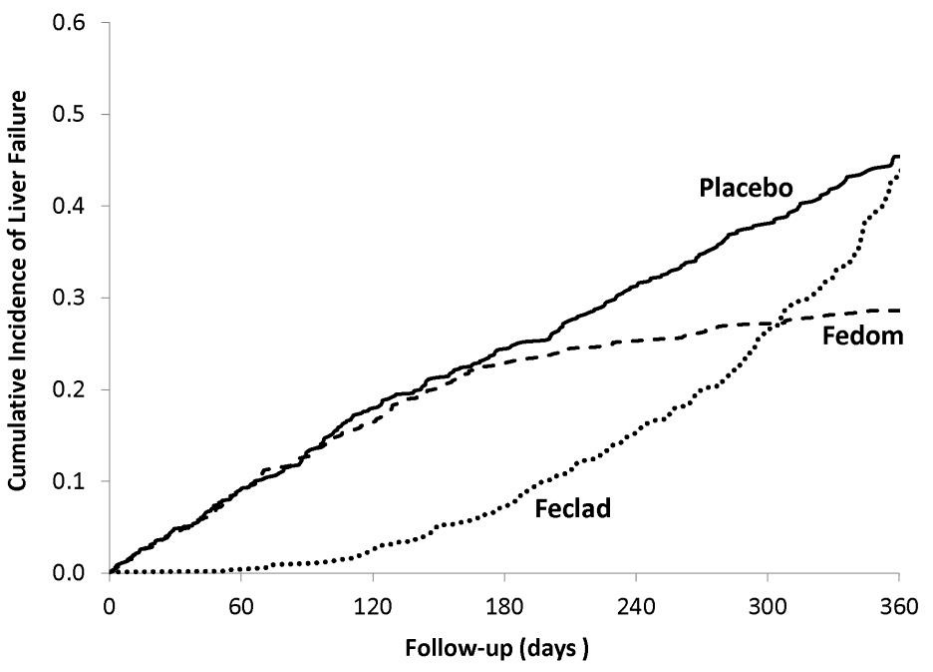
Cumulative Incidence of Liver Failure for the Hypothetical Data Cumulative incidence of liver failure as estimated by the Kaplan–Meier approach for the three treatment groups of the hypothetical three-arm 1-year trial of 3,000 patients with the (imaginary) iron overload syndrome.

While the trial was intended to follow all patients for 1 year, 60% were censored before that time. Thus, the mean follow-up was 7 months, during which there were 324 liver failures in the placebo group, compared with 230 and 238 in the Fedom and Feclad groups, respectively. Figure 1 displays the results from this trial by presenting the cumulative incidence curves (the reverse of the Kaplan–Meier curves) of liver failure for the three treatment groups over the 1-year follow-up. We now apply to the data from this trial the different techniques used by the studies with problematic results to calculate the incidence of the outcome and the corresponding NNT.

### NNT: Using the Simple Proportion?

Several trials have used the simple proportion of patients with the outcome to compute the NNT. Only if all patients are followed for the full study period does the simple proportion equal the cumulative incidence of the outcome at 1 year as computed by the Kaplan–Meier approach. However, when the follow-up times vary, as is generally the case with most trials, the simple proportion is not a valid estimate of the cumulative incidence and can thus lead to erroneous and misleading values of the NNT.

Table 1 shows the NNT calculations from the hypothetical trial in iron overload syndrome. First, using the cumulative incidence of liver failure after 1 year of treatment, estimated from the Kaplan–Meier curves (Figure 1), it shows that the number needed to treat for 1 year with Fedom is six to prevent one liver failure over that time, while for Feclad the corresponding number needed to be treat is 77 over 1 year. In contrast, using the simple proportion of patients with liver failure, which does not account for the varying follow-up times, leads to values of the number needed to treat for 1 year of 11 with Fedom and 12 with Feclad to prevent one liver failure over that time. The differences with the NNT properly based on the Kaplan–Meier approach are significant.

**Table 1:** Comparison Between NNT Computed from the Simple Proportion of Liver Failure with That from More Proper Cumulative Incidence Based on the Kaplan–Meier Approach for the Hypothetical Three-arm 1-Year Trial of 3,000 Patients with the (Imaginary) Iron Overload Syndrome.

			Using Kaplan–Meier Approach		Using Simple Proportion	
Treatment	Number of Patients	Number with Liver Failure	1-Year Cumulative Incidence	Number Needed to Treat	1-Year Cumulative Incidence	Number Needed to Treat
Placebo	1000	324	0.454		0.324	

Fedom	1000	230	0.286	6	0.230	11

Feclad	1000	238	0.441	77	0.238	12

Note that the importance of the Kaplan–Meier approach is not as crucial in studies where the follow-up is short and mostly complete, in which case the simple proportions and Kaplan–Meier cumulative incidence are practically equal.^19^,^20^ However, as the follow-up times become more variable, the simple proportion can result in distorted values of the NNT.^16^,^17^,^21^,^22^

In the trial of nucleoside analogues against herpes simplex virus type 2 (HSV-2), the NNT calculation of 38 persons with recurrent genital herpes treated with valacyclovir for 1 year to prevent one case of HSV-2 infection was also based on the simple proportion of patients with HSV-2 infection.^17^ It was calculated as 1/[(27/741)–(14/743)]=57 from the 240-day data, which was then extrapolated to a year by multiplying by 240/365 to arrive at the “NNT” of 38 for 1 year. Instead, the Kaplan–Meier values of cumulative incidence of HSV-2 infection at day 240 are 4.3% versus 2.1%, giving a NNT of 45 for 240 days of treatment. In the trial of zoledronic acid in premenopausal women with endocrine-responsive early breast cancer, the authors used the simple proportion of patients whose disease progressed to compute the NNT plainly as 1/[(83/904)–(54/899)]=31.^16^ This is despite the fact that the Kaplan–Meier curves were estimated and provided and that follow-up varied extensively between patients.

### NNT: Using the Incidence Rate?

Another measure that has been used to quantify the incidence of the outcome in calculating the NNT has been the incidence rate as a way to account for varying follow-up times. The incidence rate is computed as the number of patients with the outcome divided by the total amount of person-time generated by the follow-up of the study patients. Using this, some authors have computed the NNT as the inverse of the difference between the incidence rates for the two groups under study. However, here again, this can lead to incorrect values of the NNT.

Table 2 displays the NNT calculations from the hypothetical trial using the incidence rate compared with the proper cumulative incidence estimates. Using the incidence rate of liver failure per patient per year leads to values of the number needed to treat for 1 year of six with Fedom, and five with Feclad, to prevent one liver failure over that time. Here, the contrast is particularly striking for Feclad, where the NNT was 77 at 1 year using the proper Kaplan–Meier approach.

**Table 2:** Comparison between NNT Computed from the Incidence Rate of Liver Failure Per Patient-Year with That from More Proper Cumulative Incidence Based on the Kaplan–Meier Approach for the Hypothetical Three-arm 1-Year Trial of 3,000 Patients with the (Imaginary) Iron Overload Syndrome.

				Using Kaplan–Meier Approach		Using Simple Rate Per Patient-year	
Treatment	Number of Patients	Number of Patient-years	Number with Liver Failure	1-Year Cumulative Incidence	Number Needed to Treat	Incidence Rate Per Patient Per Year	Number Needed to Treat
Placebo	1000	549.8	324	0.454		0.589	

Fedom	1000	576.2	230	0.286	6	0.399	6

Feclad	1000	615.7	238	0.441	77	0.387	5

Several studies have improperly used the incidence rate approach in computing the NNT.^18^,^23^–^28^ An example is from a trial of 1,801 frail elderly adults randomized to a hip protector or to a control group to assess the risk of hip fracture, with varying follow-up times (mean 1.1 years).^23^ The incidence rate of hip fracture in the hip protector group was 21.3 per 1,000 person-years compared with 46.0 in the control group. The resulting reported “number needed to treat for one year to prevent one hip fracture was 41 persons” was based on these incidence rates rather than the cumulative incidence at 1 year. The paper in fact provided the Kaplan–Meier curves for the cumulative incidence of hip fracture, which indicate a 1-year cumulative incidence of 5.0% for the hip-protector group and 2.1% for the control group, corresponding to a NNT of 35 patients needing to be treated for 1 year to prevent one hip fracture, rather than the reported 41.

Similarly, in the Collaborative Atorvastatin Diabetes Study (CARDS), the authors used incidence rates to report that “27 patients would need to be treated for 4 years to prevent one (major cardiovascular) event.”^27^ However, the Kaplan–Meier curves for the cumulative incidence of a major cardiovascular event result in a NNT value closer to 20 patients at 4 years.^27^

Lastly, the previously mentioned trial of 3,845 very elderly hypertensives randomized to a diuretic or placebo also used incidence rates to compute the NNT of 94 treated for 2 years to prevent one stroke.^18^ The Kaplan–Meier curves for the cumulative incidence of stroke indicate a 2-year cumulative incidence of stroke of 2.2% for diuretic treatment and 3.8% for placebo, corresponding to a NNT of 63 patients needing to be treated for 2 years to prevent one stroke, rather than the miscalculated 94 patients.

### NNT: Using Meta-analyses

The meta-analysis of the 22 trials of the anticholinergic tiotropium that involved over 23,000 COPD patients reported a NNT of 16 patients “over one year” with tiotropium to prevent one exacerbation.^13^ These trials involved different study durations, varying from 3 to 48 months. However, the calculation of the NNT involved all 22 trials, using the proportion of patients with an exacerbation over the pooled data, irrespective of the study duration. Indeed, 37.7% of the tiotropium patients had an exacerbation, compared with 44.2% on placebo, proportions based on a mix of short-term (3 months) and long-term (48 months) trials. Nevertheless, the reported NNT of 16 referred to the time period of treatment as “one year.”

To evaluate the NNT for 1 year of treatment, the meta-analysis could have restricted its analysis exclusively to the 1-year trials. Indeed, for the six 1-year studies, the proportion of patients with a COPD exacerbation is 37.4% of patients on tiotropium compared with 44.2% on placebo, leading to a NNT over 1 year of 15, which coincidentally is practically equal to the reported NNT of 16 based on all 22 trials of variable duration. Note, however, that NNT is 72 for the 3-month trials and 250 for the 48-month trials.^15^

Lastly, it is important to note that even for the 1-year trials, the follow-up times likely varied between patients, so that the simple proportions used can be inaccurate to compute the NNT, as shown in Table 1. Thus, in this case, one should seek Kaplan–Meier estimates that account for variable follow-up times. Moreover, one could also use data from the longer-term trials, the 4-year trial for instance, identifying the 1-year cumulative incidence values from the Kaplan–Meier estimates that span the 4 years of the study.

### NNT in Benefit–Risk Evaluation

The NNT can also be useful in evaluating the balance between a risk and benefit of a drug. Indeed, the number of patients that need to be treated to prevent an outcome of the disease can be compared to the number needed to treat to induce a patient having a harmful side-effect. For example, the NNT was useful in weighting the benefit of inhaled corticosteroids in preventing COPD exacerbations against their risk of inducing pneumonia.^29^,^30^ It was a particularly important question since pneumonias are much less frequent than COPD exacerbations, so that one is tempted to assess benefit versus risk simply on the basis of the frequency of these outcomes, rather than the drug effects.^29^ The NNT approach revealed, however, using the net effect of inhaled corticosteroids, that the risk of inducing pneumonia may outweigh the benefit of preventing exacerbations, particularly over the longer term, even if pneumonia is much less frequent.^29^,^31^ Of course, such use of the NNT in a benefit–risk assessment must also make sure that outcomes of similar importance are being compared, such as avoiding the comparison between mild COPD exacerbations that are easily treated in the outpatient setting versus pneumonias that require extended hospitalization and possibly intensive care.

Another example is the previously mentioned meta-analysis of low-dose aspirin use, where prevention of mortality was assessed against causing gastrointestinal bleeding.^3^ Using the pooled data from six trials, the number needed to treat with low-dose aspirin to prevent one death from any cause was 67, while 100 needed to be treated to induce one non-fatal gastrointestinal tract bleeding. Here again, however, this meta-analysis included trials of varying durations, which can introduce bias in the NNT when not properly accounted for, particularly if the risks or benefits vary with duration of aspirin use. Moreover, the NNT values do not refer to a specific duration of treatment with aspirin.

In contrast, an analysis pooling data from four trials to assess the benefit–risk of rivaroxaban versus enoxaparin for the prevention of venous thromboembolism (VTE) after total hip or knee arthroplasty properly used the Kaplan–Meier curves to compute the NNTs.^32^ These were measured at specific time points, namely 70 days after total hip surgery and 47 days after knee arthroplasty, for the benefit outcome of VTE or death, versus the risk outcome of bleeding.

## CONCLUSION

The number needed to treat is a simple and appealing measure of the impact of a treatment that has been, since its publication over 25 years ago, increasingly used in the reporting of study results. It provides the number of patients that need to be treated to prevent the disease outcome in one patient, over a given time period. However, despite and perhaps because of its simplicity, the NNT is often miscalculated. Generally, the incorrect computations arise from the inappropriate consideration of time.

Indeed, trials generally result in varying follow-up times so that the calculation of the NNT inherently requires that the cumulative incidence of the outcome be used in its computation. We note, however, that many studies either use simple proportions or incidence rate per patient-time, rather than Kaplan–Meier curves, thus not properly accounting for varying follow-up times. In our hypothetical trial with varying follow-up times, we noted that using the incorrect simple proportion changed the NNT of a treatment from 77 to 12, or from 6 to 11. As well, using the incorrect incidence rate changed the NNT of a treatment from 77 to 5.

Another issue is the tendency of some studies to extrapolate the NNT time horizon beyond the study time-period. For example, the trial evaluating a hip protector to prevent hip fractures was a 2-year study.^23^ However, the incidence rate of hip fracture per year was first converted to a rate per 5-year period and used to compute a 5-year NNT, i.e. “the number needed to treat for five years to prevent one hip fracture was 8 persons.” Such extrapolation of data from a 2-year study to a 5-year horizon can be problematic as the treatment effect can easily change over time. Indeed, 8 patients being treated for 5 years is not necessarily the same as 40 patients treated for 1 year or 20 patients treated for 2 years, which is precisely what such extrapolation implies. Our hypothetical trial data shows clearly in the figure that treatment effects compared to placebo at 6 months are vastly different than those at 1 year.

These miscalculations and misinterpretations of the NNT are, alas, still very current, even after 25 years of existence.^4^,^33^ A survey of papers published in 2009 in four major medical journals found no problem with the calculation of the NNT when the studies involved simple designs with fixed follow-up times, where the simple proportion equals the cumulative incidence.^34^ However, in the studies involving varying follow-up times, 60% did not compute the NNT correctly.^34^

Some issues were not discussed in this paper, such as the role of random error in the calculation of the NNT. It is of course important to attach a *P* value or a confidence interval to the NNT, so that the role of chance and the size of the study are reflected in the calculation. Computing the NNT for a non-significant treatment effect will result in an apparent anomaly in the confidence interval—it will include negative values for the NNT, which will reflect a range of NNT values where the study treatment is inferior to the comparison treatment.^35^ Including confidence intervals becomes useful when the NNT is used to compare different studies with different power or study size, such as meta-analyses and single trials. In addition, it is useful to note that variations of the concept of the NNT have been proposed. Examples include the number needed to harm (NNH) when dealing with an adverse rather than beneficial effect of treatment, the number remaining to be treated, and the number needed to screen.^36^,^37^

In all, as the “number needed to treat” becomes increasingly used to evaluate treatments, proper calculations are crucial to avoid distorting health care economic calculations and public health evaluations. The appropriate use of the cumulative incidence function over time will avoid important biases and provide accurate estimates of this appealing measure of treatment impact.

## Abbreviations

COPD: chronic obstructive pulmonary disease
HSV-2: herpes simplex virus HSV type 2
IOS: iron overload syndrome
NNT: number needed to treat
VTE: venous thromboembolism.
